# Supplementary material for: Scalability of a singing-based intervention for postpartum depression in Denmark and Romania: protocol for a single-arm feasibility study
Source: BMJ Open. 2022 Dec 13;12(12):e063420. doi: 10.1136/bmjopen-2022-063420 (PMC9748965; doi:10.1136/bmjopen-2022-063420)
Supplement: Supplementary data [file bmjopen-2022-063420supp001.pdf]

Informed consent form: Research managers

You are being invited to participate in a research study titled 'Singing for maternal low mood'. This study is being run by the World Health Organization Regional Office for Europe, University College London, and the Cluj Cultural Centre.

The purpose of this study is to test the feasibility of the introduction of arts and health interventions in different cultural and linguistic contexts.

If you agree to participate in the study you will be asked to complete some evaluation materials, and attend a weekly class for 10 weeks. Each class will take one hour. There will be focus groups and interviews after the intervention has taken place, which will each take 1-2 hours.

Consent

I would like to ask for your consent to some specific points. Please indicate if you agree with the following points:

	Please tick
You confirm that you I have read and understood the participant information sheet	
Your participation is completely voluntary	
All your answers will be used for scientific research to investigate the ease of rolling out arts and health interventions in different cultural contexts and potential policy changes that results from this research	
Your answers will be stored securely; however, personal information will be anonymized (surveys) or pseudonymized (focus groups and interviews)	
Your answers gathered in this study will be shared anonymously with relevant researchers and agencies	
You agree to the inclusion of your answers in the analysis in accordance with the information provided here.	
You are aware that the analysis, and possibly some of your answers, will be published in anonymous form.	
You can stop participating at any time. Your data will be anonymised, and data used up to the point of withdrawal will still be used	

If you would like more information about the inclusion of your answers in the analysis and publication, I am happy to provide you with this information.

Please contact me when research results are released ☐

Signature:

Date:
