# Supplementary material for: Scalability of a singing-based intervention for postpartum depression in Denmark and Romania: protocol for a single-arm feasibility study
Source: BMJ Open. 2022 Dec 13;12(12):e063420. doi: 10.1136/bmjopen-2022-063420 (PMC9748965; doi:10.1136/bmjopen-2022-063420)
Supplement: Author's manuscript [file bmjopen-2022-063420.draft_revisions.pdf]

BMJ Open is committed to open peer review. As part of this commitment we make the peer review history of every article we publish publicly available.

When an article is published we post the peer reviewers' comments and the authors' responses online. We also post the versions of the paper that were used during peer review. These are the versions that the peer review comments apply to.

The versions of the paper that follow are the versions that were submitted during the peer review process. They are not the versions of record or the final published versions. They should not be cited or distributed as the published version of this manuscript.

BMJ Open is an open access journal and the full, final, typeset and author-corrected version of record of the manuscript is available on our site with no access controls, subscription charges or pay-per-view fees (http://bmjopen.bmj.com).

If you have any questions on BMJ Open's open peer review process please email info.bmjopen@bmj.com

BMJ Open

Scalability of a singing for postpartum depression intervention in Denmark and Romania: protocol for a single-arm feasibility study

Journal:	*BMJ Open*
Manuscript ID	bmjopen-2022-063420
Article Type:	Protocol
Date Submitted by the Author:	08-Apr-2022

Complete List of Authors:	Warran, Katey; University College London, Research Department of Behavioural Science and Health<br>Smith, Calum; World Health Organization Regional Office for Europe, Behavioural and Cultural Insights Unit<br>Ugron, Hanna; Centrul Cultural Clujean<br>Frøkjær Carstens, Louise; Region Midtjylland, Den Kreative Skole<br>Zbranca, Rarita; Centrul Cultural Clujean<br>Ottow, Mikkel; Region Midtjylland<br>Blaga, Oana ; Babeș-Bolyai University, Center for Health Policy and Public Health<br>Lund Ladegaard , Nicolai ; Aarhus University, Department of Clinical Medicine<br>Davis, Rachel; King's College London, Centre for Implementation Science<br>Fancourt, Daisy; University College London Research Department of Epidemiology and Public Health, Department of Behavioural Science and Health<br>Fietje, Nils; World Health Organization Regional Office for Europe, Behavioural and Cultural Insights Unit

Keywords:	PUBLIC HEALTH, Depression & mood disorders < PSYCHIATRY, MENTAL HEALTH

SCHOLARONE™
Manuscripts

Scalability of a singing for postpartum depression intervention in Denmark and Romania: protocol for a single-arm feasibility study

Katey Warran[1], Calum Smith[2] (joint first authors)*

Hanna Ugron[3], Louise Frøkjær Carstens[4], Rarita Zbranca[3], Mikkel Ottow[5], Oana Blaga[6], Nicolai Lund Ladegaard[7], Rachel Davis[8], Daisy Fancourt[1], Nils Fietje[2]

1. Research Department of Behavioural Science and Health, University College London, London, UK
2. Behavioural and Cultural Insights Unit, World Health Organization Regional Office for Europe, Denmark
3. Centrul Cultural Clujean, Cluj-Napoca, Romania
4. Den Kreative Skole, Region Midtjylland (Central Denmark Region), Denmark
5. Region Midtjylland (Central Denmark Region), Denmark
6. Center for Health Policy and Public Health, College of Political, Babe☐-Bolyai University, Cluj-Napoca, Romania
7. Department of Clinical Medicine, Aarhus University, Denmark
8. Centre for Implementation Science, King's College London, London, UK

*Correspondence to: Dr Katey Warran, University College London, k.warran@ucl.ac.uk

For peer review only

For peer review only

Scalability of a singing for postpartum depression intervention in Denmark and Romania: protocol for a single-arm feasibility study

ABSTRACT

Introduction: Although Postpartum depression (PPD) affects around one in seven women globally, there is currently no complete treatment solution. There is a long history of the benefits of singing for maternal mental health, and promising research exists showing the clinical effectiveness of group singing in particualr. Group singing interventions are being scaled up to support new mother's in the UK, but we do not know if such an intervention may benefit women in different cultural contexts. This protocol focuses on new research exploring the feasibility of implementation and perceived impact of a 10-week group singing intervention for new mothers in Romania and Denmark eliciting signs of PPD.

Methods and analysis: Data will be collected from up to 48 women with a score ≥10 on the Edinburgh Postnatal Depression Scale (EPDS) participating in a 10-week group singing intervention in Denmark or Romania, as well as a range of different project stakeholders. The singing classes will take place in person and be facilitated by professional singing leaders. Feasibility of implementation will be analysed through qualitative data (e.g., focus groups, interviews) and quantitative data (e.g., the Feasibility of Intervention Measure [FIM]). Perceived impact will be explored via surveys that

include mental health measures (EPDS, MSPSS, WHO-5) from singing intervention participants (at weeks 1, 6, 10) and focus groups. Descriptive statistics, repeated measures ANOVAs, and ANCOVAs will be used to analyse quantitative data. Framework method and thematic analysis will be used to analyse qualitative data.

Ethics and dissemination: The National ethics committees in Romania [IRB-PH Protocol #2021-211217-012] and Denmark [case number 1-10-72-274-21] have approved the study. An application to the central Ethical Review Committee at the World Health Organisation has been submitted. Study results will be disseminated by reports published by the WHO Regional Office for Europe, peer-reviewed publications, and at conferences and events.

STRENGTHS AND LIMITATIONS OF THIS STUDY

- First study exploring how a clinically effective singing intervention from the UK is adapted to meet local needs in Romania and Denmark.
- First singing intervention for new mother's experiencing PPD symptoms implemented in Cluj, Romania, as part of a research project.
- First study to create detailed guidance and best practice related to how arts and health interventions can be implemented in and scaled to different national and cultural contexts.
- Comprehensive range of implementation measures collected to explore feasibility of implementation.
- The study does not include a control group, therefore it will not be possible to make generalizable claims.

INTRODUCTION

Postpartum (also called postnatal) depression (PPD) is a type of depression that can occur during the first year of having a baby [1]. PPD affects around one in seven women globally [2], and it can have significant impact on mental health (and consequently mental health services), even developing into suicide if left untreated [1,3,4]. Symptoms include depressive mood, reduced interest in daily activities, insomnia, agitation, guilt, and fatigue, all of which can also affect a mother's relationship with their child [2]. Notably, it has been suggested that depressed mothers exhibit two interaction styles of 'withdrawn' or 'intrusive' which may adversely affect babies due to inadequate stimulation and arousal modulation [5]. In the longer-term, this can also adversely affect a child's

For peer review only

behaviour [6] and academic performance [7]. Therefore, identifying ways to deliver holistic healthcare that can provide psychosocial support is particularly important for this group.

Treatment for PPD

There is no complete treatment solution for PPD. Pharmacological treatment shows promise (e.g., Brexanolone) but uptake and adherence are low, with treatment often delayed [8,9]. Whilst psychological therapies have been shown to improve mood, the benefits are short-term and not superior to spontaneous remission [10,11]. Additionally, there can be stigma attached to receiving treatment, and mothers can find it hard to make time to attend them [12].

In recent years, there has been a burgeoning interest in how social, arts, and community group-based activities can support mental health, quality of life, and wellbeing, alongside increased interest in finding cost-effective evidence-based interventions that can be embedded into healthcare pathways, such as via social prescribing schemes [13–16]. Group-based activities have been shown to prompt social, psychological, and behavioural responses that are linked-in with improved mental and physical health [17]. In the context of support for new mothers, mother-baby groups that include play activities have been shown to relax mothers, improve social interaction, and provide personal fulfilment, with those who join in pregnancy also displaying a reduction in depressive symptoms [18]. However, singing groups may be able to provide greater support for depressive symptoms than play activities [19].

Singing for maternal mental health

There is a long history of the benefits of singing for maternal mental health. In anthropology and evolutionary theory, it has been argued that lullabies and mother-infant singing evolved from motherese (a style of exaggerated speech between mother and baby) which was used to improve bonding and reassure babies [20,21]. Studies exploring regular singing between mother and baby have shown that singing can improve mother-infant bonding in the first nine months following birth [22]. These findings provide strong grounds to explore the clinical effects of singing sessions for new mothers. In a randomised controlled trial called 'Music and Motherhood' comparing the effects of 10-week singing and play programmes for 134 mothers with symptoms of PPD, it was found that singing reduced symptoms by 38% and led to a significantly faster improvement in symptoms for those with moderate–severe PPD symptoms. As part of the same trial, qualitative research also supported these findings, showing that reduced symptoms were facilitated through fostering a

For peer review only

functional emotional response rooted in the needs of new motherhood, including providing 'me time' for mothers and 'tools' for calming and bonding with babies [23].

Music and Motherhood and the SHAPER study

The 'Music and Motherhood' trial comparing the effects of singing and play interventions for PPD was led from 2015-17 by the Centre for Performance Science (a joint research centre that sits between the Royal College of Music and Imperial College, London) with support from University College London in the UK [24]. Following the research, the programme was developed into practice for the first time and expanded from 2017 onwards by an organisation called Breathe Arts Health Research as part of their 'Melodies for Mums' programme. The team embedded the intervention in support services in South London (Lambeth and Southwark) and later nationally as an online intervention during the COVID-19 pandemic. In order to explore the effectiveness of the intervention on a larger scale in London, the four-year 'Scaling-Up Health-Arts Programmes: Implementation and Effectiveness Research-Postnatal Depression' (SHAPER-PND) study was launched in 2020 and is ongoing [25].

The importance of culturally-sensitive psychosocial interventions

Although the SHAPER-PND study is looking to explore the impact of Music and Motherhood on a larger scale, it is limited to the UK. We therefore do not know if this intervention may benefit women in different cultural and linguistic contexts. Given the complexity of arts interventions as moderated by their local environments and involving multiple components [17,26], the first step to implementing such an intervention in a new setting is understanding how it might need to change to meet local needs. Gaining this understanding requires a level of cultural competence to frame information and deliver interventions that are sensitive to cultural values, beliefs, and attitudes towards mental health conditions [27]. Therefore, there is an important gap in the literature regarding researching how an intervention needs to be changed to meet health challenges in contexts beyond which it may have been initially designed.

This protocol

This feasibility study explores how to implement the Music and Motherhood intervention in Denmark and Romania. Both of these countries present a strong rationale for implementation.

In Romania, data on PPD is scarce and mental health literacy of postpartum depression is poor [28]. For example, out of 112 pregnant women in their 3rd trimester interviewed in clinical settings in 2019,

For peer review only - http://bmjopen.bmj.com/site/about/guidelines.xhtml

52% reported not knowing what PPD is, whereas 43% considered that they could never develop PPD [29]. The limited available research shows that up to 24% of new mothers could be affected by PPD [30]. The prevalence of PPD in Denmark has been found to be between 5.5% [31] and 7.5% [32]. National reports on available interventions targeting PPD in Denmark stress the need for early and sensitive care and services. In particular, it has been highlighted that community group-based interventions can reduce feelings of shame and increase empowerment [33]. However, there is little research on the benefits of group singing interventions for this population.

Our primary aim is to evaluate the feasibility of implementation in these new cultural contexts, and our secondary aim is to evaluate the perceived impact of the intervention on mental health and wellbeing for intervention participants. The study is funded by the Nordic Culture Fund [project number 24479], with further support from the Wellcome Trust [219425/Z/19/Z], Region Midtjylland (Denmark) and the Cluj Culture Centre (Romania), and delivered by the WHO Regional Office for Europe in partnership with University College London.

METHODS AND ANALYSIS

Setting

The singing intervention will be implemented in community centres in Denmark and Romania. The study is being conducted in partnership with Den Kreative Skole in Region Midtjylland (Central Denmark Region) in Denmark, and Cluj Culture Centre in Cluj, Transylvania in Romania. Staff from these organisations will lead the delivery, data collection, and analyses for the study in their respective countries.

There will be two 10-week singing programmes in each country, delivered by professional singing leaders. The implementation team and singing leaders will receive international training from Breathe Arts Health Research. The spaces within which the intervention will be run will have suitable space for 8-12 prams, sufficient baby changing facilities, and will be easily accessible using public transport. The locations will not be disclosed in recruitment and marketing due to the potential stigma attached to having PPD. In Romania, due to the low uptake of COVID-19 vaccination, the intervention will also be delivered in a semi-outdoor setting to ensure good ventilation. In both countries, participants will be asked to take a rapid antigen test ahead of attending each week, and tests will be supplied at no cost to the participants, if needed.

For peer review only

Patient and Public Involvement (PPI)

This protocol was developed and discussed with two PPI groups – one in Denmark and one in Romania. The PPI groups consist of mothers with lived experience, academic experts, and mental health professionals. The groups plan to meet a further 2 times throughout the study to guide the project's delivery, including supporting the development of musical content for the singing sessions.

Participants

Data will be collected from different project stakeholders (coined the 'implementation team' for this protocol) to examine how the intervention is delivered. This will include four groups:

1. Strategic ***managers/partners*** (n=7) involved in planning and overseeing the intervention delivery and success (e.g., members of the management committee from Region Midtjylland, Cluj Culture Centre, and Den Creative Skole who meet regularly with WHO/Europe and UCL for planning meetings)
2. Project ***managers/partners*** (n=7) involved in coordinating and managing the on-the-ground delivery of the intervention (e.g., project coordinators working at Cluj Culture Centre and Region Midtjylland)
3. ***Referrers*** (n≈8) (such as health nurses in Denmark or other health/NGO professionals in Romania) who refer participants to be included in the intervention. To be recruited.
4. ***Singing leaders*** (n=3) who will lead on the practical and creative delivery of the intervention. One in Denmark, two in Romania.

All managers/partners and singing leaders will need to speak English sufficiently enough to read the information sheet and give informed consent. Referrers and singing leaders will be able to participate in Romanian or Danish. All stakeholders must be aged over 18 to participate.

Data will also be collected from our singing intervention participants to explore the perceived impact of the singing classes. The aim is to include two groups of 8-12 participants in each country, a total of 32-48 participants in total across both Denmark and Romania. The inclusion criteria are women who:

- score ≥10 on the Edinburgh Postnatal Depression Scale (EPDS) (collected upon screening);
- are up to 40 weeks post-birth with an infant aged 0-9 months;
- are aged 18 or over; and

For peer review only

- have the capacity to give informed consent.

Potential singing intervention participants who cannot speak Danish, Romanian, Hungarian, or English to a level that is sufficient to read and understand the information sheets and consent forms for the study (either through reading or being read to) will be excluded, as will those under the age of 18 or those who have infants not aged 0-9 months.

Recruitment

Implementation team

All stakeholders involved in the delivery and running of the intervention will be invited to participate. Strategic and project managers/partners who are already involved in the delivery of the project will be sent an email outlining what participation in the study will involve. Referrers will be approached within countries by on-the-ground teams either in person or digitally, where the study will be explained, and they will have the option to join the research. The singing leaders will be informed of the research when they are employed to lead the classes.

Denmark intervention participants

The recruitment strategy in Denmark will follow a referral process whereby health nurses in the municipal health authority in Central Denmark Region will screen for eligibility for the intervention and, if consented, participants will be referred directly into the singing intervention. This recruitment process started in Spring 2022 and is anticipated to end in late spring/early summer 2022.

Romania intervention participants

The recruitment strategy in Romania will centre around printed and digital marketing and communications activities from the Cluj Cultural Centre. This will include dissemination of printed materials through community facilitators, adverts on local radio stations, and social media. The team will also contact gynaecological clinics (private and public), family physicians, and non-governmental organizations (NGOs) that support mothers after they give birth to share information about the project. In addition to circulating printed and digital materials, project team members will attend relevant groups and clinics (with permission from relevant stakeholders) to speak to potential participants face-to-face about the project. This recruitment process started in spring 2022 and is anticipated to end in early summer 2022.

For peer review only

For participants who request to join the intervention, but who are not eligible (see inclusion criteria), we will aim to offer one free singing workshop session as a recognition of their interest in being involved and be referred to other interventions and/or activities that may be of interest (e.g., given a list of free arts programmes, local choir groups).

The singing intervention

The starting point for the design and structure of the singing sessions was the UK Music and Motherhood intervention [24]. Informed by the UK model, it is anticipated that the classes will happen on a weekly basis for 8 weeks, last one hour, and include mothers sitting in a circle with their babies to learn songs together. There will be time to socialise before and after the class, and it is expected that the group will learn culturally diverse songs and sometimes use small instruments [24]. However, a key aim of our study is to explore how this (UK) intervention may need to change to meet local needs. The study teams will work with our PPI groups to tailor the intervention to new contexts and receive guidance and advice from Breathe Arts Health Research, UK in the form of implementation team and singing lead training sessions. The singing leader will bring their own ideas for content to discuss with the PPI group, and be trained to respond to participants' needs throughout the study, documenting any changes made so that it is possible to understand how and why the intervention changed throughout processes of delivery.

From current feedback from PPI groups in Romania and Denmark, it is expected that songs included in the classes will cover a range of genres and will not be tied to a specific language, but rather feature phonetic noises and percussive sounds. This is to ensure that, even in groups involving mothers from minority groups (Hungarian, Roma mothers), who do not share the same first language with the other Romanian mothers, no individuals wil be disadvantaged. In addition we will have a Romanian and a Hungarian speaking music leader, to reduce any language barrier.

The classes will run from late spring to late-summer 2022. The two classes in each country will have some overlap, with the first tranche of mothers beginning their participation in the intervention slightly earlier than the second tranche. This is to ensure that all the classes can take place outside in warm months if they need to due to high incidence of COVID-19.

DATA COLLECTION

Feasibility of implementation methods

For peer review only - http://bmjopen.bmj.com/site/about/guidelines.xhtml

Table 1 shows an overview of the data collection methods used to meet our primary aim of evaluating the feasibility of implementation, with the key groups and individuals who will be approached listed in the columns.

Table 1: Data collection methods to evaluate the feasibility of implementation of Music and Motherhood

Data collection method[1] (described in detail below)	Strategic managers/ partners	Project managers/ partners	Referrers	Singing leader	Intervention participants
Implementation team focus group	x	x	x		
Implementation team short survey	x	x	x	x	
Implementation team semi-structured interview				x	x
Track record document	x				
Screening and attendance log		x			x
Active ingredients worksheet				x	

[1] Topic guides informed by Soukup et al. [AIM] [42] and the Scaling-up Health-Arts Programmes: Implementation and Effectiveness Research (SHAPER) implementation protocols [1-2-1 interviews and focus groups] [25]

For peer review only

These implementation measures are informed by frameworks from implementation science including:

- The Reach Effectiveness Adoption Implementation Maintenance (RE-AIM) framework [34];
- Proctor et al.'s (2011) outcomes for implementation research taxonomy [35];
- The Medical Research Council's (MRC) guidance on carrying out process evaluations of complex interventions [36]; and
- Warran et al.'s (2022) Ingredients iN ArTs in hEalth (INNATE) Framework [26]

The data collection methods informed by these frameworks are described in more detail below:

Implementation team focus group

Implementation focus groups will be conducted with three different groups (one focus group for each group): strategic managers/partners, project managers/partners, and referrers. The topic guide for the group will ask questions to assess the following about the intervention: acceptability, appropriateness, feasibility, adoption, sustainability, unintended consequences, implementation strategies, contextual factors, success of any training delivered, and key learnings from the project. The focus groups will take place post-intervention and are expected to last 60-90 minutes.

Implementation team short survey

A short survey containing three validated measures of 4-items each (Acceptability of Intervention Measure [AIM], Intervention Appropriateness Measure [IAM], and Feasibility of Intervention Measure [FIM]) will be distributed to strategic managers/partners, project managers/partners, referrers, and the singing leader to complete post-intervention. The survey is anticipated to take approximately 5 minutes.

Implementation 1-2-1 interview

An interview will be carried out with the singing leader and with a selection of participants (n=4-5, the first volunteers will be selected) who have taken part in the intervention. The topic guide for the interviews will ask questions to assess the following: acceptability, appropriateness, feasibility, fidelity of receipt (participants only), adoption, sustainability (singing leader only), unintended consequences, contextual factors (singing leader only), and success of training delivered (singing leader only). The interviews will take place post-intervention and are expected to last 60 minutes each.

For peer review only

Track record document

A document is being created and maintained by WHO/Europe to record meeting minutes of management committee meetings (with strategic managers/partners) and meetings with partners in Denmark and Romania. This document will serve as a resource to document our implementation processes, how decisions were made throughout, and to explore key learnings from the project, as well as to assess fidelity of delivery.

Screening and attendance logs

Logs will be created and maintained by project managers/coordinators to document the number of people who were screened and how many of these individuals went on to participate in the intervention. A record will also be kept of intervention attendance by the singing leader, including whether any participants drop out throughout the 10-week programme. This will help us to assess the reach of the project (i.e., whether the intended audiences came into contact with the intervention, and how).

Active ingredients worksheet

To aid the process of tracking and understanding any adaptations made to the intervention throughout the process of implementation, the Ingredients iN ArTs in hEalth (INNATE) Framework will be used [37]. This framework puts forward a comprehensive mapping of 139 active ingredients (the components of an intervention that prompt the mechanisms of action underlying potential health outcomes) which can be used to describe, in depth, the key aspects of an intervention that may be important to health. The framework will be used to identify, and reflect on, intervention active ingredients at the planning stage of the intervention (i.e., the week before the classes are implemented in each country) and then again after the intervention is complete (i.e., after week 10). This tool will therefore support with assessing fidelity of delivery through documenting adaptations to the intervention that occur organically, as well as provide a document that could be used to support with replication of the intervention into other countries in the future.

Perceived impact of intervention methods

Survey

Intervention participants will be invited to complete a short (5 minute) quantitative survey at three time points throughout study participation (weeks 1, 6, and 10). These surveys have been kept

For peer review only

purposely short to try and keep the process simple and easy for participants to engage in. See Table 2.

Table 2: Quantitative measures to evaluate the perceived impact of the intervention on mental health and wellbeing for singing participants

Outcome	Type	Measure	When
Postpartum depression	Quantitative	Edinburgh Postnatal Depression Scale – EPDS	Weeks 1, 6, and 10
Social support	Quantitative	Multidimensional Scale of Perceived Social Support – MSPSS	Weeks 1, 6, and 10
Wellbeing	Quantitative	The World Health Organisation Five Well-Being Index – WHO-5	Weeks 1, 6, and 10

Focus group

All intervention participants will be invited to join one focus group at the end of the 10-week programme (one focus group per singing group). The groups will explore subjective experiences of the weekly sessions such as in relation to what participants enjoyed/didn't enjoy, any challenges that occurred, and the perceived impact of the classes on PPD symptoms, social relationships, and identity. The focus group is anticipated to last 60-90 minutes.

Optional reflective journal entry

Study participants will be given the option to complete a journal entry at the end of the singing programme to reflect on anything they feel they have been unable to share at other data collection points.

In addition to the above qualitative and quantitative data, we will also collect sociodemographic data (location, age, ethnicity, educational attainment).

See figure 1 for a flow diagram of all of our data collection time points and measures.

Figure 1: Study flow chart

For peer review only

<Insert study flow chart here – see end of paper>

DATA ANALYSES

Quantitative

Data will be analysed using Stata and/or MASPSS. Descriptive statistics will be used to analyse the implementation short survey (AIM, IAM, FIM), and repeated measures ANOVAs will be used to explore changes to mental health and wellbeing across time for our singing intervention participants (within-subject changes). ANCOVAs will additionally explore whether these changes are moderated by participants' sociodemographic backgrounds.

Qualitative

Framework Method will be used to organize and analyse the qualitative data collected from intervention participants relating to implementation (i.e., one-on-one interviews) [38]. The study teams across Denmark and Romania, with support from University College London, will develop a framework for use that is underpinned by approaches from implementation science, as previously outlined (e.g., RE-AIM, Proctor's taxonomy). The framework will be used to construct qualitative themes from the data that are relevant to our primary aim of evaluating implementation.

Drawing upon guidance from Braun et al. [39,40] a reflexive thematic approach will be taken to analyse and evaluate experiences of the singing group and its perceived impact (i.e., from the focus groups and journal entries). This will involve generating initial codes, searching for and reviewing themes, and then defining and naming themes. An inductive approach will be taken, working from the bottom-up, generating or drawing out knowledge from the data through examination of what is meaningful for the participants, thereby placing participant experiences at the heart of the analysis [41]. This will allow us to evaluate perceived changes to mental health and wellbeing. Qualitative analysis software will be used to analyse and organise qualitative data.

Ethics and dissemination

Dissemination

The findings will initially be written up in a *project implementation report*, published by the WHO Regional Office for Europe and made publicly available. The aim of this report will be to share our processes and learnings with commissioners, relevant organizations, policymakers, and healthcare

For peer review only

institutions so that it can be used in the future to provide guidance and best practice related to how arts and health interventions can be implemented in and scaled to different national and cultural contexts. Findings will also be published in peer-reviewed journals and shared at conferences, meetings, and events, including webinars run by WHO/Europe.

Ethics

The National ethics committees in Romania [IRB-PH Protocol #2021-211217-012] and Denmark [case number 1-10-72-274-21] have approved the study. An application to the central Ethical Review Committee at the World Health Organisation has been submitted.

Authors' contributions:

All authors listed made contributions to the conception and design of the study protocol. CS and KW led equally on the drafting of the protocol report for this publication, with specific feedback from NF. KW led on the design of the methodology for the data collection, and NF led on the overall supervision of the project, including funding acquisition. All authors have approved the final protocol for publication.

Acknowledgements

The authors would like to thank Dr Rosie Perkins (Royal College of Music) and Dr Jill Sonke (University of Florida) for their peer review on the WHO/Europe ethics application that informed this protocol. They would also like to thank Yvonne Farquharson, Hannah Dye and Lorna Greenwood from Breathe Arts Health Research for the training provided and insight shared in view of their Breathe Melodies for Mums programme.

Funding statement:

This work is supported by the Nordic Culture Fund [project number 24479], with further support from The Wellcome Trust [219425/Z/19/Z], Region Midtjylland (Denmark) and the Cluj Culture Centre (Romania).

Competing interests statement: No competing interests.

For peer review only

References

1. Dennis C-L, Dowswell T. Psychosocial and psychological interventions for preventing postpartum depression. Cochrane Database Syst Rev [Internet]. 2013 Feb 28 [cited 2019 Oct 8];(2). Available from: http://doi.wiley.com/10.1002/14651858.CD001134.pub3
2. Mughal S, Azhar Y, Siddiqui W. Postpartum Depression. StatPearls [Internet]. 2021 Jul 2 [cited 2022 Feb 17]; Available from: https://www.ncbi.nlm.nih.gov/books/NBK519070/
3. Lindahl V, Pearson JL, Colpe L. Prevalence of suicidality during pregnancy and the postpartum. Arch Women's Ment Heal [Internet]. 2005 Jun 11 [cited 2019 Oct 8];8(2):77–87. Available from: http://www.ncbi.nlm.nih.gov/pubmed/15883651
4. Warran K, Ward J. The National Women and Children's Creative Health Handbook: Wellbeing by Design [Internet]. 2020 [cited 2022 Mar 21]. Available from: https://www.improvingme.org.uk/media/1151/womenandchildrenscreativehealthhandbook.pdf
5. Welch MG, Hofer MA, Brunelli SA, Stark RI, Andrews HF, Austin J, et al. Family nurture intervention (FNI): Methods and treatment protocol of a randomized controlled trial in the NICU. BMC Pediatr. 2012 Feb 7;12.
6. Fihrer I, McMahon CA, Taylor AJ. The impact of postnatal and concurrent maternal depression on child behaviour during the early school years. J Affect Disord [Internet]. 2009 Dec 1 [cited 2019 Oct 14];119(1–3):116–23. Available from: https://www.sciencedirect.com/science/article/pii/S0165032709000986
7. Hay DF, Pawlby S, Sharp D, Asten P, Mills A, Kumar R. Intellectual Problems Shown by 11-year-old Children Whose Mothers Had Postnatal Depression. J Child Psychol Psychiatry [Internet]. 2001 Oct [cited 2019 Oct 14];42(7):871–89. Available from: http://www.ncbi.nlm.nih.gov/pubmed/11693583
8. Scarff JR. Use of Brexanolone for Postpartum Depression. Innov Clin Neurosci [Internet]. 2019 Nov 1 [cited 2022 Feb 25];16(11–12):32. Available from: /pmc/articles/PMC7006866/
9. Fitelson E, Kim S, Baker AS, Leight K. Treatment of postpartum depression: clinical, psychological and pharmacological options. Int J Womens Health [Internet]. 2011 [cited 2022 Feb 25];3(1):1. Available from: /pmc/articles/PMC3039003/
10. Cooper PJ, Murray L, Wilson A, Romaniuk H. Controlled trial of the short- and long-term effect of psychological treatment of post-partum depression. Impact on maternal mood. Br J Psychiatry. 2003 May 1;182(MAY):420–7.
11. Morrell C, Warner R, Slade P, Dixon S, Walters S, Paley G, et al. Psychological interventions for postnatal depression: cluster randomised trial and economic evaluation. The PoNDER trial. Health Technol Assess (Rockv) [Internet]. 2009 Jun [cited 2019 Oct

For peer review only

8];13(30):iii–iv, xi–xiii, 1–153. Available from: http://www.ncbi.nlm.nih.gov/pubmed/19555590

12. McCarthy M, McMahon C. Acceptance and Experience of Treatment for Postnatal Depression in a Community Mental Health Setting. http://dx.doi.org/101080/07399330802089172 [Internet]. 2008 Jul [cited 2022 Mar 21];29(6):618–37. Available from: https://www.tandfonline.com/doi/abs/10.1080/07399330802089172

13. Fancourt D, Finn S. What is the evidence on the role of the arts in improving health and well-being? A scoping review [Internet]. WHO Regional Office for Europe (Health Evidence Network synthesis report no. 67); 2019 [cited 2020 Jan 24]. ix + 133 pp. Available from: http://www.euro.who.int/en/publications/abstracts/what-is-the-evidence-on-the-role-of-the-arts-in-improving-health-and-well-being-a-scoping-review-2019

14. The All-Party Parliamentary Group on Arts H and W. Creative Health: The Arts for Health and Wellbeing [Internet]. London; 2017 [cited 2017 Oct 27]. Available from: http://www.artshealthandwellbeing.org.uk/appg-inquiry/Publications/Creative_Health_Inquiry_Report_2017.pdf

15. Fancourt D, Warran K, Aughterson H. Evidence summary for policy: The role of arts in improving health and wellbeing. 2020.

16. Fancourt D, Bhui K, Chatterjee H, Crawford P, Crossick G, Denora T, et al. Social, cultural and community engagement and mental health: cross-disciplinary, co-produced research agenda. [cited 2022 Feb 17]; Available from: https://doi.org/

17. Fancourt D, Aughterson H, Finn S, Walker E, Steptoe A. How leisure activities affect health: A review and multi-level theoretical framework of mechanisms of action using the lens of complex adaptive systems science. Lancet Psychiatry. 2020;

18. Salo SJ, Flykt M, Mäkelä J, Biringen Z, Kalland M, Pajulo M, et al. The effectiveness of Nurture and Play: A mentalisation-based parenting group intervention for prenatally depressed mothers. Prim Heal Care Res Dev. 2019;

19. Fancourt D, Perkins R. Effect of singing interventions on symptoms of postnatal depression: three-arm randomised controlled trial. Br J Psychiatry [Internet]. 2018 Feb 9 [cited 2018 Sep 7];212(02):119–21. Available from: http://www.ncbi.nlm.nih.gov/pubmed/29436333

20. Falk D. Prelinguistic evolution in early hominins: Whence motherese? Behav Brain Sci. 2004 Aug;27(4):491–503.

21. Fancourt D, Perkins R. Associations between singing to babies and symptoms of postnatal depression, wellbeing, self-esteem and mother-infant bond. Public Health. 2017 Apr 1;145:149–52.

22. Persico G, Antolini L, Vergani P, Costantini W, Nardi MT, Bellotti L. Maternal singing of lullabies during pregnancy and after birth: Effects on mother–infant bonding and on

For peer review only

newborns' behaviour. Concurrent Cohort Study. Women and Birth [Internet]. 2017 Aug [cited 2019 Oct 16];30(4):e214–20. Available from: http://www.ncbi.nlm.nih.gov/pubmed/28169158

23. Perkins R, Yorke S, Fancourt D. How group singing facilitates recovery from the symptoms of postnatal depression: a comparative qualitative study. 2018 [cited 2019 Mar 22]; Available from: https://doi.org/10.1186/s40359-018-0253-0

24. Fancourt D, Perkins R. Creative interventions for symptoms of postnatal depression: A process evaluation of implementation. Arts Heal [Internet]. 2019;11(1):38–53. Available from: http://doi.org/10.1080/17533015.2017.1413398

25. Estevao C, Bind R, Fancourt D, Sawyer K, Dazzan P, Sevdalis N, et al. SHAPER-PND trial: clinical effectiveness protocol of a community singing intervention for postnatal depression. BMJ Open [Internet]. 2021 Nov 1 [cited 2022 Feb 17];11(11):e052133. Available from: https://bmjopen.bmj.com/content/11/11/e052133

26. Warran K, Burton A, Fancourt D. What are the active ingredients of 'arts in health' activities? Development of the INgredients iN ArTs in hEalth (INNATE) Framework. Wellcome Open Res 2022 710 [Internet]. 2022 Jan 11 [cited 2022 Feb 17];7:10. Available from: https://wellcomeopenresearch.org/articles/7-10

27. Napier D, Ancarno C, Butler B, Calabrese J, Chater A, Chatterjee H, et al. Culture and health [Internet]. Vol. 384, thelancet.com. 2014 [cited 2022 Feb 25]. Available from: http://dx.doi.org/10.1016/

28. Swami V, Vintila M, Goian C, Tudorel O, Bucur V. Mental Health Literacy of Maternal and Paternal Postnatal Depression in a Community Sample of Romanian Adults. https://doi.org/101037/ipp0000130 [Internet]. 2020 Apr 9 [cited 2022 Mar 21];9(3):147–58. Available from: https://econtent.hogrefe.com/doi/abs/10.1037/ipp0000130

29. Dimitriu M, Stoian AP, Rosu G, Dan A, Caimacan A, Zygouropoulos N, et al. Postpartum Depression-a serious, dangerous, disabling, extremely frequent condition that is almost completely ignored in Romania. Mediterr J Clin Psychol [Internet]. 2020 [cited 2022 Mar 21];8(1). Available from: https://doi.org/10.6092/2282-1619/mjcp-2413

30. Enatescu VR, Bernad E, Gluhovschi A, Papava I, Romosan R, Palicsak A, et al. Perinatal characteristics and mother's personality profile associated with increased likelihood of postpartum depression occurrence in a Romanian outpatient sample. http://dx.doi.org/103109/0963823720161149802 [Internet]. 2016 May 4 [cited 2022 Mar 21];26(3):212–9. Available from: https://www.tandfonline.com/doi/abs/10.3109/09638237.2016.1149802

31. Nielsen D, Videbech P, Hedegaard M, Dalby J, Secher NJ. Postpartum depression: identification of women at risk. BJOG [Internet]. 2000 [cited 2022 Mar 21];107(10):1210–7. Available from: https://pubmed.ncbi.nlm.nih.gov/11028570/

For peer review only

32. Pommerencke L, Pant S, Ammitzbøll J, Laursen B, Pedersen T. Forældres psykiske tilstand i barnets første le- veår og betydning for barnets udvikling og trivsel. Temarapport fra Databasen Børns Sundhed for børn født i 2018 og skoleåret 2019/20. København; 2021.

33. Det Nationale Forsknings- og Analysecenter for Velfærd (VIVE). Indsatser i forhold til fødselsdepression - En litteraturgennemgang af eksisterende viden [Internet]. 2022. Available from: https://www.vive.dk/media/pure/17003/6377310

34. Harden S, Smith M, Ory M, Smith-Ray R, Estabrooks P, Glasgow R. RE-AIM in Clinical, Community, and Corporate Settings: Perspectives, Strategies, and Recommendations to Enhance Public Health Impact. Front public Heal [Internet]. 2018 Mar 22 [cited 2021 Sep 1];6(MAR). Available from: https://pubmed.ncbi.nlm.nih.gov/29623270/

35. Proctor E, Silmere H, Raghavan R, Hovmand P, Aarons G, Bunger A, et al. Outcomes for implementation research: conceptual distinctions, measurement challenges, and research agenda. Adm Policy Ment Health [Internet]. 2011 Mar [cited 2021 Sep 1];38(2):65–76. Available from: https://pubmed.ncbi.nlm.nih.gov/20957426/

36. Moore G, Audrey S, Barker M, Bond L, Bonell C, Hardeman W, et al. Process evaluation of complex interventions: UK Medical Research Council (MRC) guidance. 2015.

37. Warran K, Burton A, Fancourt D. What are the active ingredients of 'arts in health' activities? Development of the INgredients iN ArTs in hEalth (INNATE) Framework. Wellcome Open Research. https://doi.org/10.12688/wellcomeopenres.17414.1

38. Nicola K Gale, Heath G, Cameron E, Rashid S, Redwood S. Using the framework method for the analysis of qualitative data in multidisciplinary health research. BMC Med Res Methodol. 2013;13(117):1–8.

39. Braun V, Clarke V. Using thematic analysis in psychology. Qual Res Psychol. 2006;3(2):77–101.

40. Braun V, Clarke V, Hayfield N, Terry G. Thematic Analysis. In: Handbook of Research Methods in Health Social Sciences. Singapore: Springer; 2019. p. 843–60.

41. Williamon A, Ginsborg J, Perkins R, Waddell G. Performing Music Research: Methods in Music Education, Psychology, and Performance Science. Oxford: Oxford University Press;

42. Soukup T, Hull L, Smith EL, Healey A, Bakolis I, Amiel SA, et al. Effectiveness-implementation hybrid type 2 trial evaluating two psychoeducational programmes for severe hypoglycaemia in type 1 diabetes: implementation study protocol. BMJ Open [Internet]. 2019 Nov 1 [cited 2021 Sep 1];9(11):e030370. Available from: https://bmjopen.bmj.com/content/9/11/e030370

For peer review only

List of figures

Figure 1: Study flow chart. Diagram showing data collection time points and measures collected for singing intervention participants and the implementation team.

For peer review only

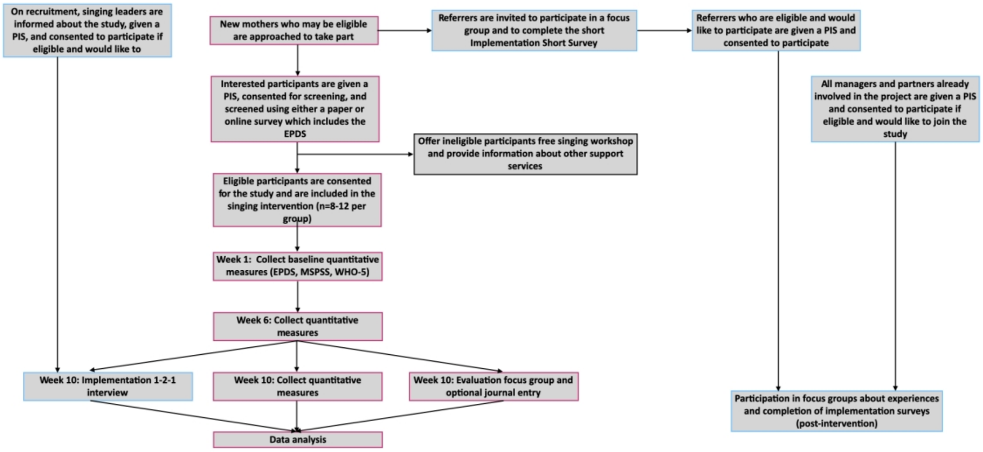

Figure 1: Study flow chart

677x381mm (72 x 72 DPI)

BMJ Open

Scalability of a singing-based intervention for postpartum depression in Denmark and Romania: protocol for a single-arm feasibility study

Journal:	*BMJ Open*
Manuscript ID	bmjopen-2022-063420.R1
Article Type:	Protocol
Date Submitted by the Author:	20-Oct-2022

Complete List of Authors:	Warran, Katey; University College London, Research Department of Behavioural Science and Health<br>Smith, Calum; World Health Organization Regional Office for Europe, Behavioural and Cultural Insights Unit; University of Oxford, Nuffield Department of Population Health<br>Ugron, Hanna; Centrul Cultural Clujean<br>Frøkjær Carstens, Louise; Region Midtjylland, Den Kreative Skole<br>Zbranca, Rarita; Centrul Cultural Clujean<br>Ottow, Mikkel; Region Midtjylland<br>Blaga, Oana ; Babeș-Bolyai University, Center for Health Policy and Public Health<br>Lund Ladegaard , Nicolai ; Aarhus University, Department of Clinical Medicine<br>Davis, Rachel; King's College London, Centre for Implementation Science<br>Fancourt, Daisy; University College London Research Department of Epidemiology and Public Health, Department of Behavioural Science and Health<br>Fietje, Nils; World Health Organization Regional Office for Europe, Behavioural and Cultural Insights Unit

**Primary Subject Heading**:	Public health
Secondary Subject Heading:	Mental health
Keywords:	PUBLIC HEALTH, Depression & mood disorders < PSYCHIATRY, MENTAL HEALTH

SCHOLARONE™ Manuscripts

Scalability of a singing-based intervention for postpartum depression in Denmark and Romania: protocol for a single-arm feasibility study

Katey Warran[1], Calum Smith[2,9] (joint first authors)*

Hanna Ugron[3], Louise Frøkjær Carstens[4], Rarita Zbranca[3], Mikkel Ottow[5], Oana Blaga[6], Nicolai Lund Ladegaard[7], Rachel Davis[8], Daisy Fancourt[1], Nils Fietje[2]

1. Research Department of Behavioural Science and Health, University College London, London, UK
2. Behavioural and Cultural Insights Unit, World Health Organization Regional Office for Europe, Denmark
3. Centrul Cultural Clujean, Cluj-Napoca, Romania
4. Den Kreative Skole, Region Midtjylland (Central Denmark Region), Denmark
5. Region Midtjylland (Central Denmark Region), Denmark
6. Center for Health Policy and Public Health, College of Political, Babe☐-Bolyai University, Cluj-Napoca, Romania
7. Department of Clinical Medicine, Aarhus University, Denmark
8. Centre for Implementation Science, King's College London, London, UK
9. Nuffield Department of Population Health, University of Oxford, Oxford, UK

*Correspondence to:

Dr Katey Warran

k.warran@ucl.ac.uk

For peer review only

For peer review only

ABSTRACT

Introduction: Postpartum depression (PPD) affects around one in seven women globally, with these women in need of non-pharmaceutical treatment strategies. There is a long history of the benefits of singing for maternal mental health, and promising research exists showing the clinical effectiveness of group singing. Group singing interventions are being scaled up to support new mothers in the UK, but we do not know if such an intervention may benefit women in different cultural contexts. This protocol focuses on exploring the feasibility of implementation and perceived impact of a 10-week group singing intervention for new mothers in Romania and Denmark eliciting signs of PPD.

Methods and analysis: Data will be collected from up to 48 women with a score ≥10 on the Edinburgh Postnatal Depression Scale (EPDS) participating in a 10-week group singing intervention in Denmark or Romania, as well as a range of project stakeholders. The singing classes will take place in person and be facilitated by professional singing leaders. Feasibility of implementation will be analysed through qualitative data (e.g., focus groups, interviews) and quantitative data (e.g., the

For peer review only - http://bmjopen.bmj.com/site/about/guidelines.xhtml

Feasibility of Intervention Measure [FIM]). Perceived impact will be explored via surveys that include mental health measures (EPDS, MSPSS, WHO-5) from singing intervention participants (at weeks 1, 6, 10) and focus groups. Descriptive statistics, repeated measures ANOVAs, and ANCOVAs will be used to analyse quantitative data. Framework method and thematic analysis will be used to analyse qualitative data.

Ethics and dissemination: The national ethics committees in Romania [IRB-PH Protocol #2021-211217-012] and Denmark [case number 1-10-72-274-21] have approved the study, as has the central Ethical Review Committee at the World Health Organisation [ERC.0003714]. All participants will be required to provide informed consent. Results will be disseminated by reports published by the WHO Regional Office for Europe, peer-reviewed publications, and at conferences.

STRENGTHS AND LIMITATIONS OF THIS STUDY

- Comprehensive range of implementation measures collected to explore feasibility of implementation.
- Uses mixed-methods to explore the implementation of a complex singing intervention from multiple perspectives.
- The study does not include a control group, therefore it will not be possible to make generalizable claims in relation to the quantitative impact of the intervention.
- Includes data collection from mothers and study managers/partners, strategic managers/parthers, and referrers, to provide broad perspectives on the feasibility of implementation.
- Includes an embedded qualitative component to explore the subjective impact of the intervention.

INTRODUCTION

Postpartum (also called postnatal) depression (PPD) is a type of depression that can occur during the first year of having a baby [1]. PPD affects around one in seven women globally [2], and it can have significant impact on mental health (and consequently mental health services), even developing into suicide if left untreated [1,3,4]. Symptoms include depressive mood, reduced interest

For peer review only

in daily activities, insomnia, agitation, guilt, and fatigue, all of which can also affect a mother's relationship with their child [2]. Notably, it has been suggested that depressed mothers exhibit two interaction styles of 'withdrawn' or 'intrusive' which may adversely affect babies due to inadequate stimulation and arousal modulation [5]. In the longer-term, this can also adversely affect a child's behaviour [6] and academic performance [7]. Therefore, identifying ways to deliver holistic healthcare that can provide psychosocial support is particularly important for this group.

Treatment for PPD

There is no complete treatment solution for PPD. Pharmacological treatment shows promise (e.g., Brexanolone) but uptake and adherence are low, with treatment often delayed [8,9]. Whilst psychological therapies have been shown to improve mood, the benefits are short-term and not superior to spontaneous remission [10,11]. Additionally, there can be stigma attached to receiving treatment, and mothers can find it hard to make time to attend them [12].

In recent years, there has been a burgeoning interest in how social, arts, and community group-based activities can support mental health, quality of life, and wellbeing, alongside increased interest in finding cost-effective evidence-based interventions that can be embedded into healthcare pathways, such as via social prescribing schemes [13–16]. Group-based activities have been shown to prompt social, psychological, and behavioural responses that are linked-in with improved mental and physical health [17]. In the context of support for new mothers, mother-baby groups that include play activities have been shown to relax mothers, improve social interaction, and provide personal fulfilment, with those who join in pregnancy also displaying a reduction in depressive symptoms [18]. However, singing groups may be able to provide greater support for depressive symptoms than play activities [19].

Singing for maternal mental health

There is a long history of the benefits of singing for maternal mental health. In anthropology and evolutionary theory, it has been argued that lullabies and mother-infant singing evolved from motherese (a style of exaggerated speech between mother and baby) which was used to improve bonding and reassure babies [20,21]. Studies exploring regular singing between mother and baby have shown that singing can improve mother-infant bonding in the first nine months following birth [22]. These findings provide strong grounds to explore the clinical effects of singing sessions for new mothers. In a randomised controlled trial called 'Music and Motherhood' comparing the effects

For peer review only

of 10-week singing and play programmes for 134 mothers with symptoms of PPD, it was found that singing reduced symptoms by 38% and led to a significantly faster improvement in symptoms for those with moderate–severe PPD symptoms. As part of the same trial, qualitative research also supported these findings, showing that reduced symptoms were facilitated through fostering a functional emotional response rooted in the needs of new motherhood, including providing 'me time' for mothers and 'tools' for calming and bonding with babies [23].

Music and Motherhood and the SHAPER study

The 'Music and Motherhood' trial comparing the effects of singing and play interventions for PPD was led from 2015-17 by the Centre for Performance Science (a joint research centre that sits between the Royal College of Music and Imperial College, London) with support from University College London in the UK [24]. Following the research, the programme was developed into practice for the first time and expanded from 2017 onwards by an organisation called Breathe Arts Health Research as part of their 'Melodies for Mums' programme. The team embedded the intervention in support services in South London (Lambeth and Southwark) and later nationally as an online intervention during the COVID-19 pandemic. In order to explore the effectiveness of the intervention on a larger scale in London, the four-year 'Scaling-Up Health-Arts Programmes: Implementation and Effectiveness Research-Postnatal Depression' (SHAPER-PND) study was launched in 2020 and is ongoing [25].

The importance of culturally-sensitive psychosocial interventions

Although the SHAPER-PND study is looking to explore the impact of Music and Motherhood on a larger scale, it is limited to the UK. We therefore do not know if this intervention may benefit women in different cultural and linguistic contexts. Given the complexity of arts interventions as moderated by their local environments and involving multiple components [17,26], the first step to implementing such an intervention in a new setting is understanding how it might need to change to meet local needs. Gaining this understanding requires a level of cultural competence to frame information and deliver interventions that are sensitive to cultural values, beliefs, and attitudes towards mental health conditions [27]. Therefore, there is an important gap in the literature regarding researching how an intervention needs to be changed to meet health challenges in contexts beyond which it may have been initially designed.

This protocol

For peer review only

For peer review only - http://bmjopen.bmj.com/site/about/guidelines.xhtml

This feasibility study explores how to implement the Music and Motherhood intervention in Denmark and Romania. Both of these countries present a strong rationale for implementation.

In Romania, data on PPD is scarce and mental health literacy of postpartum depression is poor [28]. For example, out of 112 pregnant women in their 3rd trimester interviewed in clinical settings in 2019, 52% reported not knowing what PPD is, whereas 43% considered that they could never develop PPD [29]. The limited available research shows that up to 24% of new mothers could be affected by PPD [30]. The prevalence of PPD in Denmark has been found to be between 5.5% [31] and 7.5% [32]. National reports on available interventions targeting PPD in Denmark stress the need for early and sensitive care and services. In particular, it has been highlighted that community group-based interventions can reduce feelings of shame and increase empowerment [33]. However, there is little research on the benefits of group singing interventions for this population.

Our primary aim is to evaluate the feasibility of implementation in these new cultural contexts, and our secondary aim is to evaluate the perceived impact of the intervention on mental health and wellbeing for intervention participants. The study is funded by the Nordic Culture Fund [project number 24479], with further support from the Wellcome Trust [219425/Z/19/Z], Region Midtjylland (Denmark) and the Cluj Culture Centre (Romania), and delivered by Dr Nils Fietje at the WHO Regional Office for Europe (Sponsor) in partnership with University College London.

METHODS AND ANALYSIS

Setting

The singing intervention will be implemented in community centres in Denmark and Romania. The study is being conducted in partnership with Den Kreative Skole in Region Midtjylland (Central Denmark Region) in Denmark, and Cluj Culture Centre in Cluj, Transylvania in Romania. Staff from these organisations will lead the delivery, data collection, and analyses for the study in their respective countries.

There will be two 10-week singing programmes in each country, delivered by professional singing leaders. The implementation team and singing leaders will receive international training from Breathe Arts Health Research. The spaces within which the intervention will be run will have suitable space for 8-12 prams, sufficient baby changing facilities, and will be easily accessible using public

For peer review only

transport. The locations will not be disclosed in recruitment and marketing due to the potential stigma attached to having PPD. In Romania, due to the low uptake of COVID-19 vaccination, the intervention will also be delivered in a semi-outdoor setting to ensure good ventilation. In both countries, participants will be asked to take a rapid antigen test ahead of attending each week, and tests will be supplied at no cost to the participants, if needed.

Patient and public involvement

This protocol was developed and discussed with two patient and public involvement (PPI) groups – one in Denmark and one in Romania. The PPI groups consist of mothers with lived experience, academic experts, and mental health professionals. The groups plan to meet a further 2 times throughout the study to guide the project's delivery, including supporting the development of musical content for the singing sessions.

Participants

Data will be collected from different project stakeholders (coined the 'implementation team' for this protocol) to examine how the intervention is delivered. This will include four groups:

1. Strategic ***managers/partners*** (n=7) involved in planning and overseeing the intervention delivery and success (e.g., members of the management committee from Region Midtjylland, Cluj Culture Centre, and Den Creative Skole who meet regularly with WHO/Europe and University College London (UCL) for planning meetings)
2. Project ***managers/partners*** (n=7) involved in coordinating and managing the on-the-ground delivery of the intervention (e.g., project coordinators working at Cluj Culture Centre and Region Midtjylland)
3. ***Referrers*** (n≈8) (such as health nurses in Denmark or other health/NGO professionals in Romania) who refer participants to be included in the intervention. To be recruited.
4. ***Singing leaders*** (n=3) who will lead on the practical and creative delivery of the intervention. One in Denmark, two in Romania.

All managers/partners and singing leaders will need to speak English sufficiently enough to read the information sheet and give informed consent (see supplementary files 1 and 2). Referrers and singing leaders will be able to participate in Romanian or Danish. All stakeholders must be aged over 18 to participate. Project managers/partners from across both countries will engage in regular

For peer review only - http://bmjopen.bmj.com/site/about/guidelines.xhtml

bi-weekly online meetings with the strategic management team as a form of supervision, ensuring ongoing reflection on ethical practice (in relation to both delivery of the intervention to participants and ensuring staff wellbeing) and fidelity of delivery.

Data will also be collected from our singing intervention participants to explore the perceived impact of the singing classes. Based on class sizes that have been successful in the UK context [19], the aim is to include two groups of 8-12 participants in each country, a total of 32-48 participants in total across both Denmark and Romania. Of note, we did not calculate our sample size in view of determining statistical power, instead focusing on how to feasibly implement the intervention and explore its perceived impact. The inclusion criteria are women who:

- score ≥10 on the Edinburgh Postnatal Depression Scale (EPDS) (collected upon screening);
- are up to 40 weeks post-birth with an infant aged 0-9 months;
- are aged 18 or over; and
- have the capacity to give informed consent.

Potential singing intervention participants who cannot speak Danish, Romanian, Hungarian, or English to a level that is sufficient to read and understand the information sheets and consent forms for the study (either through reading or being read to) will be excluded, as will those under the age of 18 or those who have infants not aged 0-9 months. See participant consent forms in supplementary files 3 and 4. Participants can still join if they are receiving other treatment.

Recruitment

Implementation team

All stakeholders involved in the delivery and running of the intervention will be invited to participate. Strategic and project managers/partners who are already involved in the delivery of the project will be sent an email outlining what participation in the study will involve. Referrers will be approached within countries by on-the-ground teams either in person or digitally, where the study will be explained, and they will have the option to join the research. The singing leaders will be informed of the research when they are employed to lead the classes, and asked to provide informed consent for their participation. They will have the right to withdraw from the study, but it will be discussed on a case-by-case basis regarding whether ending participation in the research would also end their

For peer review only - http://bmjopen.bmj.com/site/about/guidelines.xhtml

employment, based on what stage of the study they choose to withdraw (i.e., reflecting on if there is time to recruit an alternative lead to participate in the research).

Denmark intervention participants

The recruitment strategy in Denmark will follow a referral process whereby health nurses in the municipal health authority in Central Denmark Region will screen for eligibility for the intervention and, if consented, participants will be referred directly into the singing intervention. We will also supplement this approach with reaching out to relevant local non-governmental organizations (NGOs) and mother and baby groups (online and in-person) to ensure a diverse sample. This recruitment process started in Spring 2022 and is anticipated to end in autumn 2022.

Romania intervention participants

As there is no structure in place to enable direct referral vial healthcare services, the recruitment strategy in Romania will centre around printed and digital marketing and communications activities from the Cluj Cultural Centre. This will include dissemination of printed materials through community facilitators, adverts on local radio stations, and social media. The team will also contact gynaecological clinics (private and public), family physicians, and non-governmental organizations (NGOs) that support mothers after they give birth to share information about the project. In addition to circulating printed and digital materials, project team members will attend relevant groups and clinics (with permission from relevant stakeholders) to speak to potential participants face-to-face about the project. This recruitment process started in spring 2022 and ended in early summer 2022.

In both countries, we will ensure that our recruitment approach is one founded upon values of inclusivity and diversity, seeking to ensure that all those who wish to engage can. Participants will be consented by the project manager in each respective country. For participants who request to join the intervention, but who are not eligible (see inclusion criteria), we will aim to offer one free singing workshop session as a recognition of their interest in being involved and be referred to other interventions and/or activities that may be of interest (e.g., given a list of free arts programmes, local choir groups).

The singing intervention

The starting point for the design and structure of the singing sessions was the UK Music and Motherhood intervention [24]. Informed by the UK model, it is anticipated that the classes will happen

For peer review only - http://bmjopen.bmj.com/site/about/guidelines.xhtml

on a weekly basis for 8 weeks, last one hour, and include mothers sitting in a circle with their babies to learn songs together. There will be time to socialise before and after the class, and it is expected that the group will learn culturally diverse songs and sometimes use small instruments [24]. However, a key aim of our study is to explore how this (UK) intervention may need to change to meet local needs. The study teams will work with our PPI groups to tailor the intervention to new contexts and receive guidance and advice from Breathe Arts Health Research, UK in the form of implementation team and singing lead training sessions. The singing leader will bring their own ideas for content to discuss with the PPI group, and be trained to respond to participants' needs throughout the study, documenting any changes made so that it is possible to understand how and why the intervention changed throughout processes of delivery.

From current feedback from PPI groups in Romania and Denmark, it is expected that songs included in the classes will cover a range of genres and will not be tied to a specific language, but rather feature phonetic noises and percussive sounds. This is to ensure that, even in groups involving mothers from minority groups (Hungarian, Roma mothers), who do not share the same first language with the other Romanian mothers, no individuals wil be disadvantaged. In addition we will have a Romanian and a Hungarian speaking music leader, to reduce any language barrier.

The classes will run from late spring to late-summer 2022. The two classes in each country will have some overlap, with the first tranche of mothers beginning their participation in the intervention slightly earlier than the second tranche. This is to ensure that all the classes can take place outside in warm months if they need to due to high incidence of COVID-19.

Data collection

Feasibility of implementation methods

Table 1 shows an overview of the data collection methods used to meet our primary aim of evaluating the feasibility of implementation, with the key groups and individuals who will be approached listed in the columns.

<u>Table 1: Data collection methods to evaluate the feasibility of implementation of Music and Motherhood</u>

For peer review only - http://bmjopen.bmj.com/site/about/guidelines.xhtml

**Data collection method*** (described in detail below)	**Strategic managers/ partners**	**Project managers/ partners**	**Referrers**	**Singing leader**	**Intervention participants**
Implementation team focus group	X	x	x		
Implementation team short survey	X	x	x	x	
Implementation team semi-structured interview				x	x
Track record document	X				
Screening and attendance log		x		x	
Active ingredients worksheet				x	

**Topic guides informed by Soukup et al. [AIM] [34] and the Scaling-up Health-Arts Programmes: Implementation and Effectiveness Research (SHAPER) implementation protocols [1-2-1 interviews and focus groups] [25]*

These implementation measures are informed by frameworks from implementation science including:

- The Reach Effectiveness Adoption Implementation Maintenance (RE-AIM) framework [35];
- Proctor et al.'s (2011) outcomes for implementation research taxonomy [36];
- The Medical Research Council's (MRC) guidance on carrying out process evaluations of complex interventions [37]; and

For peer review only - http://bmjopen.bmj.com/site/about/guidelines.xhtml

- Warran et al.'s (2022) Ingredients iN ArTs in hEalth (INNATE) Framework [26]

The data collection methods informed by these frameworks are described in more detail below:

Implementation team focus group

Implementation focus groups will be conducted with three different groups (one focus group for each group): strategic managers/partners, project managers/partners, and referrers. The topic guide for the group will ask questions to assess the following about the intervention: acceptability, appropriateness, feasibility, adoption, sustainability, unintended consequences, implementation strategies, contextual factors, success of any training delivered, and key learnings from the project. The focus groups will take place post-intervention and are expected to last 60-90 minutes.

Implementation team short survey

A short survey containing three validated measures of 4-items each (Acceptability of Intervention Measure [AIM], Intervention Appropriateness Measure [IAM], and Feasibility of Intervention Measure [FIM]) will be distributed to strategic managers/partners, project managers/partners, referrers, and the singing leader to complete post-intervention. The survey is anticipated to take approximately 5 minutes.

Implementation 1-2-1 interview

An interview will be carried out with the singing leader and with a selection of participants (n=4-5, the first volunteers will be selected) who have taken part in the intervention. We will also invite those who drop out to participate in a 1-2-1 interview if they would like to. The topic guide for the interviews will ask questions to assess the following: acceptability, appropriateness, feasibility, fidelity of receipt (participants only), adoption, sustainability (singing leader only), unintended consequences, contextual factors (singing leader only), adequacy of training delivered (singing leader only) and sense of preparedness (singing leader only). The interviews will take place post-intervention and are expected to last 60 minutes each.

Track record document

A document is being created and maintained by WHO/Europe to record meeting minutes of management committee meetings (with strategic managers/partners) and meetings with partners in Denmark and Romania. This document will serve as a resource to document our implementation

For peer review only - http://bmjopen.bmj.com/site/about/guidelines.xhtml

processes, how decisions were made throughout, and to explore key learnings from the project, as well as to assess fidelity of delivery.

Screening and attendance logs

Logs will be created and maintained by project managers/coordinators to document the number of people who were screened and how many of these individuals went on to participate in the intervention. A record will also be kept of intervention attendance by the singing leader, including whether any participants drop out throughout the 10-week programme. This will help us to assess the reach of the project (i.e., whether the intended audiences came into contact with the intervention, and how).

Active ingredients worksheet

To aid the process of tracking and understanding any adaptations made to the intervention throughout the process of implementation, the Ingredients iN ArTs in hEalth (INNATE) Framework will be used [38]. This framework puts forward a comprehensive mapping of 139 active ingredients (the components of an intervention that prompt the mechanisms of action underlying potential health outcomes) which can be used to describe, in depth, the key aspects of an intervention that may be important to health. The framework will be used to identify, and reflect on, intervention active ingredients at the planning stage of the intervention (i.e., the week before the classes are implemented in each country) and then again after the intervention is complete (i.e., after week 10). This tool will therefore support with assessing fidelity of delivery through documenting adaptations to the intervention that occur organically, as well as provide a document that could be used to support with replication of the intervention into other countries in the future.

Perceived impact of intervention methods

Survey

Intervention participants will be invited to complete a short (5 minute) quantitative survey at three time points throughout study participation (weeks 1, 6, and 10). Participants will be contacted via email with the surveys and followed up with email reminders to improve adherence. These surveys have been kept purposely short to try and keep the process simple and easy for participants to engage in. See Table 2.

For peer review only

For peer review only - http://bmjopen.bmj.com/site/about/guidelines.xhtml

Table 2: Quantitative measures to evaluate the perceived impact of the intervention on mental health and wellbeing for singing participants

Outcome	Type	Measure	When
Postpartum depression	Quantitative	Edinburgh Postnatal Depression Scale – EPDS	Weeks 1, 6, and 10
Social support	Quantitative	Multidimensional Scale of Perceived Social Support – MSPSS	Weeks 1, 6, and 10
Wellbeing	Quantitative	The World Health Organisation Five Well-Being Index – WHO-5	Weeks 1, 6, and 10

Focus group

All intervention participants will be invited to join one focus group at the end of the 10-week programme (one focus group per singing group). The groups will explore subjective experiences of the weekly sessions such as in relation to what participants enjoyed/didn't enjoy, any challenges that occurred, and the perceived impact of the classes on PPD symptoms, social relationships, and identity. The focus group is anticipated to last 60-90 minutes. Given the potential sensitive nature of discussing maternal mental health, focus group facilitators will receive specific training delivered by UCL on how to conduct these sessions in a sensitive way.

Optional reflective journal entry

Study participants will be given the option to complete a journal entry at the end of the singing programme to reflect on anything they feel they have been unable to share at other data collection points.

In addition to the above qualitative and quantitative data, we will also collect sociodemographic data (location, age, ethnicity, educational attainment).

See figure 1 for a flow diagram of all of our data collection time points and measures.

Data analyses

For peer review only

Quantitative

Data will be analysed using Stata and/or MASPSS. Descriptive statistics will be used to analyse the implementation short survey (AIM, IAM, FIM), and repeated measures ANOVAs will be used to explore changes to mental health and wellbeing across time for our singing intervention participants (within-subject changes). ANCOVAs will additionally explore whether these changes are moderated by participants' sociodemographic backgrounds.

Qualitative

Framework Method will be used to organize and analyse the qualitative data collected from intervention participants relating to implementation (i.e., one-on-one interviews) [39]. The study teams across Denmark and Romania, with support from University College London, will develop a framework for use that is underpinned by approaches from implementation science, as previously outlined (e.g., RE-AIM, Proctor's taxonomy). The framework will be used to construct qualitative themes from the data that are relevant to our primary aim of evaluating implementation.

Drawing upon guidance from Braun et al. [40–42] a reflexive thematic approach will be taken to analyse and evaluate experiences of the singing group and its perceived impact (i.e., from the focus groups and journal entries). This will involve generating initial codes, searching for and reviewing themes, and then defining and naming themes. An inductive approach will be taken, working from the bottom-up, generating or drawing out knowledge from the data through examination of what is meaningful for the participants, thereby placing participant experiences at the heart of the analysis [43]. This will allow us to evaluate perceived changes to mental health and wellbeing. Qualitative analysis software will be used to analyse and organise qualitative data.

Analyses will be undertaken by researchers in each country who were not part of the intervention development or any management processes. Project managers, researchers, and members of the management team will also engage in regular bi-weekly meetings throughout data collection and analyses to engage in discussion about emerging findings as a form of reflexivity on our processes, thereby improving the rigor of the study.

ETHICS AND DISSEMINATION

Ethics

For peer review only - http://bmjopen.bmj.com/site/about/guidelines.xhtml

The National ethics committees in Romania [IRB-PH Protocol #2021-211217-012] and Denmark [case number 1-10-72-274-21] have approved the study, as has the central Ethical Review Committee at the World Health Organisation [ERC.0003714]. All participants will need to provide informed consent in order to participate in the study. Consent forms for both countries are included as Supplementary Files.

Dissemination

The findings will initially be written up in a *project implementation report*, published by the WHO Regional Office for Europe and made publicly available. The aim of this report will be to share our processes and learnings with commissioners, relevant organizations, policymakers, and healthcare institutions so that it can be used in the future to provide guidance and best practice related to how arts and health interventions can be implemented in and scaled to different national and cultural contexts. Findings will also be published in peer-reviewed journals and shared at conferences, meetings, and events, including webinars run by WHO/Europe.

Data protection and confidentiality

In Denmark, all data will be stored and handled following the Danish law, specifically databeskyttelsesloven and databeskyttelsesforordningen. In Romania, data will be stored in line with local data management laws. In both countries, consent forms and identifiable data will be stored securely and only accessible by the project manager in each country. The quantitative data will be anonymised and the qualitative transcripts will be pseudonymised before being shared with researchers in each respective country for analysis. All identifiable data will be stored separately to participant demographics. Quantitative data will be collected using Redcap in Denmark and using Microsoft Teams in Romania. Any extracted data used for analysis will be stored on secure, password-protected storage systems that only the researchers doing the analysis and the project managers will have access to. Audio recordings from qualitative interviews will be deleted immediately from hand-held devices and from storage facilities as soon as transcripts have been created. All identiable information will be deleted at the end of the study. Anonymised and pseudonymised data will be stored in line with data collection policies in each country – 5 years in Denmark and 5 years in Romania. Any adverse events or unintended consequences will be discussed at management meetings and documented by NF and CS at WHO/Europe in the Track Record Document (see p.8)

For peer review only

** ** **

Contributors

All authors listed made contributions to the conception and design of the study protocol. CS and KW led equally on the drafting of the protocol report for this publication, with specific feedback from NF. KW led on the design of the methodology for the data collection, and NF led on the overall supervision of the project, including funding acquisition. HU and RZ provided specific advice on the Romanian context, and FC and MO provided advice on the Danish context. NL and OB supported with developing data protection and data handling procedures. RD provided insights on the implementation measures based on her experience of working on the UK SHAPER study, and DF provided extra supervisory support in view of developing the UK intervention. All authors have approved the final protocol for publication. The Nordic Culture Fund who funded the study have played no part in the development of this protocol.

Acknowledgements

The authors would like to thank Dr Rosie Perkins (Royal College of Music) and Dr Jill Sonke (University of Florida) for their peer review on the WHO/Europe ethics application that informed this protocol. They would also like to thank Yvonne Farquharson, Hannah Dye and Lorna Greenwood from Breathe Arts Health Research for the training provided and insight shared in view of their Breathe Melodies for Mums programme.

Funding

This work is supported by the Nordic Culture Fund [project number 24479], with further support from The Wellcome Trust [219425/Z/19/Z], Region Midtjylland (Denmark) and the Cluj Culture Centre (Romania).

Competing interests

No competing interests.

Data availability statement

Anonymised survey data from intervention participants will be deposited in a public, open access repository for use by other researchers once the study is complete. Interview and focus group data

For peer review only

is not being publicly archived for use by other researchers because the data contains information that could compromise the privacy of research participants.

References

1. Dennis C-L, Dowswell T. Psychosocial and psychological interventions for preventing postpartum depression. Cochrane Database Syst Rev [Internet]. 2013 Feb 28 [cited 2019 Oct 8];(2). Available from: http://doi.wiley.com/10.1002/14651858.CD001134.pub3
2. Mughal S, Azhar Y, Siddiqui W. Postpartum Depression. StatPearls [Internet]. 2021 Jul 2 [cited 2022 Feb 17]; Available from: https://www.ncbi.nlm.nih.gov/books/NBK519070/
3. Lindahl V, Pearson JL, Colpe L. Prevalence of suicidality during pregnancy and the postpartum. Arch Women's Ment Heal [Internet]. 2005 Jun 11 [cited 2019 Oct 8];8(2):77–87. Available from: http://www.ncbi.nlm.nih.gov/pubmed/15883651
4. Warran K, Ward J. The National Women and Children's Creative Health Handbook: Wellbeing by Design [Internet]. 2020 [cited 2022 Mar 21]. Available from: https://www.improvingme.org.uk/media/1151/womenandchildrenscreativehealthhandbook.pdf
5. Welch MG, Hofer MA, Brunelli SA, Stark RI, Andrews HF, Austin J, et al. Family nurture intervention (FNI): Methods and treatment protocol of a randomized controlled trial in the NICU. BMC Pediatr. 2012 Feb 7;12.
6. Fihrer I, McMahon CA, Taylor AJ. The impact of postnatal and concurrent maternal depression on child behaviour during the early school years. J Affect Disord [Internet]. 2009 Dec 1 [cited 2019 Oct 14];119(1–3):116–23. Available from: https://www.sciencedirect.com/science/article/pii/S0165032709000986
7. Hay DF, Pawlby S, Sharp D, Asten P, Mills A, Kumar R. Intellectual Problems Shown by 11-year-old Children Whose Mothers Had Postnatal Depression. J Child Psychol Psychiatry [Internet]. 2001 Oct [cited 2019 Oct 14];42(7):871–89. Available from: http://www.ncbi.nlm.nih.gov/pubmed/11693583
8. Scarff JR. Use of Brexanolone for Postpartum Depression. Innov Clin Neurosci [Internet]. 2019 Nov 1 [cited 2022 Feb 25];16(11–12):32. Available from: /pmc/articles/PMC7006866/
9. Fitelson E, Kim S, Baker AS, Leight K. Treatment of postpartum depression: clinical, psychological and pharmacological options. Int J Womens Health [Internet]. 2011 [cited 2022 Feb 25];3(1):1. Available from: /pmc/articles/PMC3039003/
10. Cooper PJ, Murray L, Wilson A, Romaniuk H. Controlled trial of the short- and long-term effect of psychological treatment of post-partum depression. Impact on maternal mood. Br J

For peer review only

Psychiatry. 2003 May 1;182(MAY):420–7.

11. Morrell C, Warner R, Slade P, Dixon S, Walters S, Paley G, et al. Psychological interventions for postnatal depression: cluster randomised trial and economic evaluation. The PoNDER trial. Health Technol Assess (Rockv) [Internet]. 2009 Jun [cited 2019 Oct 8];13(30):iii–iv, xi–xiii, 1–153. Available from: http://www.ncbi.nlm.nih.gov/pubmed/19555590

12. McCarthy M, McMahon C. Acceptance and Experience of Treatment for Postnatal Depression in a Community Mental Health Setting. http://dx.doi.org/101080/07399330802089172 [Internet]. 2008 Jul [cited 2022 Mar 21];29(6):618–37. Available from: https://www.tandfonline.com/doi/abs/10.1080/07399330802089172

13. Fancourt D, Finn S. What is the evidence on the role of the arts in improving health and well-being? A scoping review [Internet]. WHO Regional Office for Europe (Health Evidence Network synthesis report no. 67); 2019 [cited 2020 Jan 24]. ix + 133 pp. Available from: http://www.euro.who.int/en/publications/abstracts/what-is-the-evidence-on-the-role-of-the-arts-in-improving-health-and-well-being-a-scoping-review-2019

14. The All-Party Parliamentary Group on Arts H and W. Creative Health: The Arts for Health and Wellbeing [Internet]. London; 2017 [cited 2017 Oct 27]. Available from: http://www.artshealthandwellbeing.org.uk/appg-inquiry/Publications/Creative_Health_Inquiry_Report_2017.pdf

15. Fancourt D, Warran K, Aughterson H. Evidence summary for policy: The role of arts in improving health and wellbeing. 2020.

16. Fancourt D, Bhui K, Chatterjee H, Crawford P, Crossick G, Denora T, et al. Social, cultural and community engagement and mental health: cross-disciplinary, co-produced research agenda. [cited 2022 Feb 17]; Available from: https://doi.org/

17. Fancourt D, Aughterson H, Finn S, Walker E, Steptoe A. How leisure activities affect health: A review and multi-level theoretical framework of mechanisms of action using the lens of complex adaptive systems science. Lancet Psychiatry. 2020;

18. Salo SJ, Flykt M, Mäkelä J, Biringen Z, Kalland M, Pajulo M, et al. The effectiveness of Nurture and Play: A mentalisation-based parenting group intervention for prenatally depressed mothers. Prim Heal Care Res Dev. 2019;

19. Fancourt D, Perkins R. Effect of singing interventions on symptoms of postnatal depression: three-arm randomised controlled trial. Br J Psychiatry [Internet]. 2018 Feb 9 [cited 2018 Sep 7];212(02):119–21. Available from: http://www.ncbi.nlm.nih.gov/pubmed/29436333

20. Falk D. Prelinguistic evolution in early hominins: Whence motherese? Behav Brain Sci. 2004 Aug;27(4):491–503.

21. Fancourt D, Perkins R. Associations between singing to babies and symptoms of postnatal

For peer review only

For peer review only - http://bmjopen.bmj.com/site/about/guidelines.xhtml

depression, wellbeing, self-esteem and mother-infant bond. Public Health. 2017 Apr 1;145:149–52.

22. Persico G, Antolini L, Vergani P, Costantini W, Nardi MT, Bellotti L. Maternal singing of lullabies during pregnancy and after birth: Effects on mother–infant bonding and on newborns' behaviour. Concurrent Cohort Study. Women and Birth [Internet]. 2017 Aug [cited 2019 Oct 16];30(4):e214–20. Available from: http://www.ncbi.nlm.nih.gov/pubmed/28169158
23. Perkins R, Yorke S, Fancourt D. How group singing facilitates recovery from the symptoms of postnatal depression: a comparative qualitative study. 2018 [cited 2019 Mar 22]; Available from: https://doi.org/10.1186/s40359-018-0253-0
24. Fancourt D, Perkins R. Creative interventions for symptoms of postnatal depression: A process evaluation of implementation. Arts Heal [Internet]. 2017;11(1):38–53. Available from: http://doi.org/10.1080/17533015.2017.1413398
25. Estevao C, Bind R, Fancourt D, Sawyer K, Dazzan P, Sevdalis N, et al. SHAPER-PND trial: clinical effectiveness protocol of a community singing intervention for postnatal depression. BMJ Open [Internet]. 2021 Nov 1 [cited 2022 Feb 17];11(11):e052133. Available from: https://bmjopen.bmj.com/content/11/11/e052133
26. Warran K, Burton A, Fancourt D. What are the active ingredients of 'arts in health' activities? Development of the INgredients iN ArTs in hEalth (INNATE) Framework. Wellcome Open Res 2022 710 [Internet]. 2022 Jan 11 [cited 2022 Feb 17];7:10. Available from: https://wellcomeopenresearch.org/articles/7-10
27. Napier D, Ancarno C, Butler B, Calabrese J, Chater A, Chatterjee H, et al. Culture and health [Internet]. Vol. 384, thelancet.com. 2014 [cited 2022 Feb 25]. Available from: http://dx.doi.org/10.1016/
28. Swami V, Vintila M, Goian C, Tudorel O, Bucur V. Mental Health Literacy of Maternal and Paternal Postnatal Depression in a Community Sample of Romanian Adults. https://doi.org/101037/ipp0000130 [Internet]. 2020 Apr 9 [cited 2022 Mar 21];9(3):147–58. Available from: https://econtent.hogrefe.com/doi/abs/10.1037/ipp0000130
29. Dimitriu M, Stoian AP, Rosu G, Dan A, Caimacan A, Zygouropoulos N, et al. Postpartum Depression-a serious, dangerous, disabling, extremely frequent condition that is almost completely ignored in Romania. Mediterr J Clin Psychol [Internet]. 2020 [cited 2022 Mar 21];8(1). Available from: https://doi.org/10.6092/2282-1619/mjcp-2413
30. Enatescu VR, Bernad E, Gluhovschi A, Papava I, Romosan R, Palicsak A, et al. Perinatal characteristics and mother's personality profile associated with increased likelihood of postpartum depression occurrence in a Romanian outpatient sample. http://dx.doi.org/103109/0963823720161149802 [Internet]. 2016 May 4 [cited 2022 Mar 21];26(3):212–9. Available from:

For peer review only - http://bmjopen.bmj.com/site/about/guidelines.xhtml

https://www.tandfonline.com/doi/abs/10.3109/09638237.2016.1149802

31. Nielsen D, Videbech P, Hedegaard M, Dalby J, Secher NJ. Postpartum depression: identification of women at risk. BJOG [Internet]. 2000 [cited 2022 Mar 21];107(10):1210–7. Available from: https://pubmed.ncbi.nlm.nih.gov/11028570/
32. Pommerencke L, Pant S, Ammitzbøll J, Laursen B, Pedersen T. Forældres psykiske tilstand i barnets første le- veår og betydning for barnets udvikling og trivsel. Temarapport fra Databasen Børns Sundhed for børn født i 2018 og skoleåret 2019/20. København; 2021.
33. Det Nationale Forsknings- og Analysecenter for Velfærd (VIVE). Indsatser i forhold til fødselsdepression - En litteraturgennemgang af eksisterende viden [Internet]. 2022. Available from: https://www.vive.dk/media/pure/17003/6377310
34. Soukup T, Hull L, Smith EL, Healey A, Bakolis I, Amiel SA, et al. Effectiveness-implementation hybrid type 2 trial evaluating two psychoeducational programmes for severe hypoglycaemia in type 1 diabetes: implementation study protocol. BMJ Open [Internet]. 2019 Nov 1 [cited 2021 Sep 1];9(11):e030370. Available from: https://bmjopen.bmj.com/content/9/11/e030370
35. Harden S, Smith M, Ory M, Smith-Ray R, Estabrooks P, Glasgow R. RE-AIM in Clinical, Community, and Corporate Settings: Perspectives, Strategies, and Recommendations to Enhance Public Health Impact. Front public Heal [Internet]. 2018 Mar 22 [cited 2021 Sep 1];6(MAR). Available from: https://pubmed.ncbi.nlm.nih.gov/29623270/
36. Proctor E, Silmere H, Raghavan R, Hovmand P, Aarons G, Bunger A, et al. Outcomes for implementation research: conceptual distinctions, measurement challenges, and research agenda. Adm Policy Ment Health [Internet]. 2011 Mar [cited 2021 Sep 1];38(2):65–76. Available from: https://pubmed.ncbi.nlm.nih.gov/20957426/
37. Moore G, Audrey S, Barker M, Bond L, Bonell C, Hardeman W, et al. Process evaluation of complex interventions: UK Medical Research Council (MRC) guidance. 2015.
38. Warran K, Burton A, Fancourt D. What are the active ingredients of 'arts in health' activities? Development of the INgredients iN ArTs in hEalth (INNATE) Framework.
39. Nicola K Gale, Heath G, Cameron E, Rashid S, Redwood S. Using the framework method for the analysis of qualitative data in multidisciplinary health research. BMC Med Res Methodol. 2013;13(117):1–8.
40. Braun V, Clarke V. Using thematic analysis in psychology. Qual Res Psychol. 2006;3(2):77–101.
41. Braun V, Clarke V, Hayfield N, Terry G. Thematic Analysis. In: Handbook of Research Methods in Health Social Sciences. Singapore: Springer; 2019. p. 843–60.
42. Clarke V, Braun V. Thematic analysis: A practical guide. Braun VB, Clarke V, editors. SAGE Publications Ltd; 2022.

For peer review only - http://bmjopen.bmj.com/site/about/guidelines.xhtml

43. Williamon A, Ginsborg J, Perkins R, Waddell G. Performing Music Research: Methods in Music Education, Psychology, and Performance Science. Oxford: Oxford University Press; 2021.

FIGURE TITLE/LEGEND

Figure 1: Study flow chart

Diagram showing data collection time points and measures collected for singing intervention participants and the implementation team.

List of Supplementary Files

Supplementary File 1: Research managers consent form – Romania

Supplementary File 2: Research managers consent form Denmark

Supplementary File 3: Mothers consent form - Romania

Supplementary File 4: Mothers consent form Denmark

For peer review only

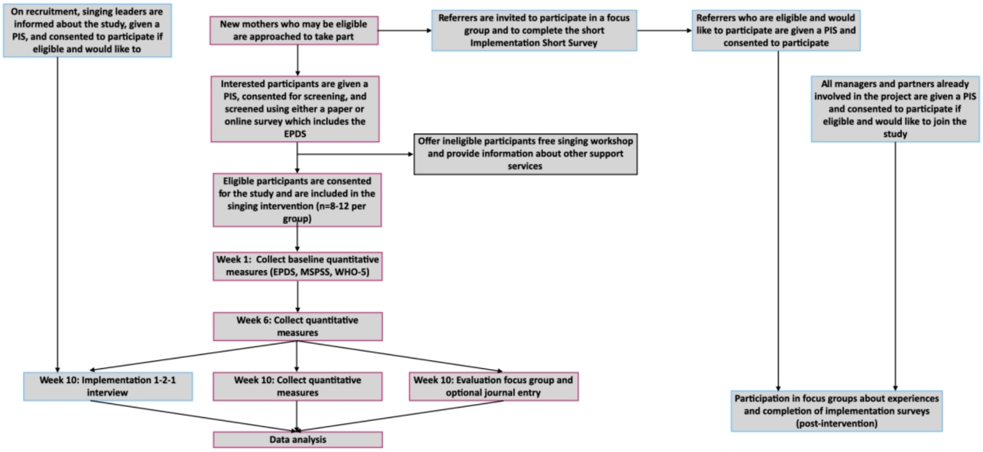

Figure 1: Study flow chart

677x381mm (72 x 72 DPI)

Informed consent form: Research managers

You are being invited to participate in a research study titled 'Singing for maternal low mood'. This study is being run by the World Health Organization Regional Office for Europe, University College London, and the Cluj Cultural Centre.

The purpose of this study is to test the feasibility of the introduction of arts and health interventions in different cultural and linguistic contexts.

If you agree to participate in the study you will be asked to complete some evaluation materials, and attend a weekly class for 10 weeks. Each class will take one hour. There will be focus groups and interviews after the intervention has taken place, which will each take 1-2 hours.

Consent

I would like to ask for your consent to some specific points. Please indicate if you agree with the following points:

	Please tick
You confirm that you I have read and understood the participant information sheet	
Your participation is completely voluntary	
All your answers will be used for scientific research to investigate the ease of rolling out arts and health interventions in different cultural contexts and potential policy changes that results from this research	
Your answers will be stored securely; however, personal information will be anonymized (surveys) or pseudonymized (focus groups and interviews)	
Your answers gathered in this study will be shared anonymously with relevant researchers and agencies	
You agree to the inclusion of your answers in the analysis in accordance with the information provided here.	
You are aware that the analysis, and possibly some of your answers, will be published in anonymous form.	
You can stop participating at any time. Your data will be anonymised, and data used up to the point of withdrawal will still be used	

If you would like more information about the inclusion of your answers in the analysis and publication, I am happy to provide you with this information.

For peer review only

Please contact me when research results are released ☐

Signature:

Date:

For peer review only

Informed consent form: Research managers

You are being invited to participate in a research study titled 'Singing for maternal low mood'. This study is being run by the World Health Organization Regional Office for Europe, University College London, and Regional Udvikling, Region Midtjylland.

The purpose of this study is to test the feasibility of the introduction of arts and health interventions in different cultural and linguistic contexts.
If you agree to participate in the study you will be asked to complete some evaluation materials. There will be focus groups and interviews after the intervention has taken place, which will each take 1-2 hours.

Consent

I would like to ask for your consent to some specific points. Please indicate if you agree with the following points:

	Please tick
You confirm that you I have read and understood the participant information sheet	
Your participation is completely voluntary	
All your answers will be used for scientific research to investigate the ease of rolling out arts and health interventions in different cultural contexts and potential policy changes that results from this research	
Your answers will be stored securely; however, personal information will be anonymized (surveys) or pseudonymized (focus groups and interviews)	
Your answers gathered in this study will be shared anonymously with relevant researchers and agencies	
You agree to the inclusion of your answers in the analysis in accordance with the information provided here.	
You are aware that the analysis, and possibly some of your answers, will be published in anonymous form.	

If you would like more information about the inclusion of your answers in the analysis and publication, I am happy to provide you with this information.
Please contact me when research results are released ☐
Signature:
Date:

For peer review only

For peer review only

Informed consent form: Mothers

You are being invited to participate in a research study titled 'Singing for maternal low mood'. This study is being run by the World Health Organization Regional Office for Europe, University College London, and the Cluj Cultural Centre

The purpose of this study is to test the feasibility of the introduction of arts and health interventions in different cultural and linguistic contexts.

If you agree to participate in the study you will be asked to complete some evaluation materials, and attend a weekly class for 10 weeks. . Each class will last one hour. There will be option to take part in focus groups to review the intervention after it has taken place. The focus group will take 1-2 hours if you choose to take part. You are also invited to keep a journal of reflections about the intervention.

Consent

I would like to ask for your consent to some specific points. Please indicate if you agree with the following points:

	Please tick
You confirm that you I have read and understood the participant information sheet	
Your participation is completely voluntary	
All your answers will be used for scientific research purposes to investigate the ease of rolling out arts and health interventions in different cultural contexts and potential policy changes that results from this research	
Your answers will be stored securely; however, personal information will be anonymized (surveys) or pseudonymized (focus groups and interviews)	
Your answers gathered in this study will be shared anonymously with relevant researchers and agencies	
You agree to the inclusion of your answers in the analysis in accordance with the information provided here.	
You are aware that the analysis, and possibly some of your answers, will be published in anonymous form.	
You can stop participating at any time. Your data will be anonymised, and data used up to the point of withdrawal will still be used	

For peer review only

If you would like more information about the inclusion of your answers in the analysis and publication, I am happy to provide you with this information.

Please contact me when research results are released ☐

Signature:

Date:

For peer review only

Informed consent form: Mothers

You are being invited to participate in a research study titled 'Singing for maternal low mood'. This study is being run by the World Health Organization Regional Office for Europe, University College London, and Regional Udvikling, Region Midtjylland.

The purpose of this study is to test the feasibility of the introduction of arts and health interventions in different cultural and linguistic contexts.

If you agree to participate in the study you will be asked to complete some evaluation materials, and attend a weekly class for 10 weeks. Each class will last one hour. There will be option to take part in focus groups to review the intervention after it has taken place. The focus group will take 1-2 hours if you choose to take part. You are also invited to keep a journal of reflections about the intervention.

Consent

I would like to ask for your consent to some specific points. Please indicate if you agree with the following points:

	Please tick
You confirm that you I have read and understood the participant information sheet	
Your participation is completely voluntary	
All your answers will be used for scientific research purposes to investigate the ease of rolling out arts and health interventions in different cultural contexts and potential policy changes that results from this research	
Your answers will be stored securely; however, personal information will be anonymized (surveys) or pseudonymized (focus groups and interviews)	
Your answers gathered in this study will be shared anonymously with relevant researchers and agencies	
You agree to the inclusion of your answers in the analysis in accordance with the information provided here.	
You are aware that the analysis, and possibly some of your answers, will be published in anonymous form.	

If you would like more information about the inclusion of your answers in the analysis and publication, I am happy to provide you with this information.

Please contact me when research results are released ☐

Signature:

Date:

For peer review only

SPIRIT

STANDARD PROTOCOL ITEMS: RECOMMENDATIONS FOR INTERVENTIONAL TRIALS

SPIRIT 2013 Checklist: Recommended items to address in a clinical trial protocol and related documents*

Section/item	ItemNo	Description	BMJ Open submission
**Administrative information**			
Title	1	Descriptive title identifying the study design, population, interventions, and, if applicable, trial acronym	Yes, page 1
Trial registration	2a	Trial identifier and registry name. If not yet registered, name of intended registry	N/A
	2b	All items from the World Health Organization Trial Registration Data Set	N/A
Protocol version	3	Date and version identifier	N/A
Funding	4	Sources and types of financial, material, and other support	Yes, pages 4 and 11
Roles and responsibilities	5a	Names, affiliations, and roles of protocol contributors	Yes, pages 1 and 11
	5b	Name and contact information for the trial sponsor	Yes, page 4
	5c	Role of study sponsor and funders, if any, in study design; collection, management, analysis, and interpretation of data; writing of the report; and the decision to submit the report for publication, including whether they will have ultimate authority over any of these activities	Yes, page 11
	5d	Composition, roles, and responsibilities of the coordinating centre, steering committee, endpoint adjudication committee, data management team, and other individuals or groups overseeing the trial, if applicable (see Item 21a for data monitoring committee)	We have a strategic management group which is detailed on page 5
**Introduction**			
Background and rationale	6a	Description of research question and justification for undertaking the trial, including summary of relevant studies (published and unpublished) examining benefits and harms for each intervention	Yes, pages 1-3
	6b	Explanation for choice of	N/A

For peer review only

		comparators	
Objectives	7	Specific objectives or hypotheses	Yes, page 4
Trial design	8	Description of trial design including type of trial (eg, parallel group, crossover, factorial, single group), allocation ratio, and framework (eg, superiority, equivalence, noninferiority, exploratory)	Yes, pages 1, 2, and 4
**Methods: Participants, interventions, and outcomes**			
Study setting	9	Description of study settings (eg, community clinic, academic hospital) and list of countries where data will be collected. Reference to where list of study sites can be obtained	Yes, pages 4-5
Eligibility criteria	10	Inclusion and exclusion criteria for participants. If applicable, eligibility criteria for study centres and individuals who will perform the interventions (eg, surgeons, psychotherapists)	Yes, page 5
Interventions	11a	Interventions for each group with sufficient detail to allow replication, including how and when they will be administered	Yes, pages 6-7
	11b	Criteria for discontinuing or modifying allocated interventions for a given trial participant (eg, drug dose change in response to harms, participant request, or improving/worsening disease)	Yes, page 9
	11c	Strategies to improve adherence to intervention protocols, and any procedures for monitoring adherence (eg, drug tablet return, laboratory tests)	Yes, page 9
	11d	Relevant concomitant care and interventions that are permitted or prohibited during the trial	Yes, page 6
Outcomes	12	Primary, secondary, and other outcomes, including the specific measurement variable (eg, systolic blood pressure), analysis metric (eg, change from baseline, final value, time to event), method of aggregation (eg, median, proportion), and time point for each outcome. Explanation of the clinical relevance of chosen efficacy and	Yes, page 9

For peer review only

		harm outcomes is strongly recommended	
Participant timeline	13	Time schedule of enrolment, interventions (including any run-ins and washouts), assessments, and visits for participants. A schematic diagram is highly recommended (see Figure)	Yes, page 6 (and study flow chart in Figure 1)
Sample size	14	Estimated number of participants needed to achieve study objectives and how it was determined, including clinical and statistical assumptions supporting any sample size calculations	Yes, page 5
Recruitment	15	Strategies for achieving adequate participant enrolment to reach target sample size	Yes, page 6
**Methods: Assignment of interventions (for controlled trials)**			
Allocation:			
Sequence generation	16a	Method of generating the allocation sequence (eg, computer-generated random numbers), and list of any factors for stratification. To reduce predictability of a random sequence, details of any planned restriction (eg, blocking) should be provided in a separate document that is unavailable to those who enrol participants or assign interventions	Study is non-randomised but we detail recruitment on page 6
Allocation concealment mechanism	16b	Mechanism of implementing the allocation sequence (eg, central telephone; sequentially numbered, opaque, sealed envelopes), describing any steps to conceal the sequence until interventions are assigned	Study is non-randomised but we detail recruitment on page 6
Implementation	16c	Who will generate the allocation sequence, who will enrol participants, and who will assign participants to interventions	Yes, page 6
Blinding (masking)	17a	Who will be blinded after assignment to interventions (eg, trial participants, care providers, outcome assessors, data analysts), and how	N/A – not blinding as this is a single-arm trial where we are primarily exploring feasibility of implementation
	17b	If blinded, circumstances under which unblinding is permissible, and procedure for revealing a participant's allocated intervention	N/A

For peer review only - http://bmjopen.bmj.com/site/about/guidelines.xhtml

		during the trial	
**Methods: Data collection, management, and analysis**			
Data collection methods	18a	Plans for assessment and collection of outcome, baseline, and other trial data, including any related processes to promote data quality (eg, duplicate measurements, training of assessors) and a description of study instruments (eg, questionnaires, laboratory tests) along with their reliability and validity, if known. Reference to where data collection forms can be found, if not in the protocol	Yes, pages 7-9
	18b	Plans to promote participant retention and complete follow-up, including list of any outcome data to be collected for participants who discontinue or deviate from intervention protocols	Yes, page 8
Data management	19	Plans for data entry, coding, security, and storage, including any related processes to promote data quality (eg, double data entry; range checks for data values). Reference to where details of data management procedures can be found, if not in the protocol	Yes, page 11
Statistical methods	20a	Statistical methods for analysing primary and secondary outcomes. Reference to where other details of the statistical analysis plan can be found, if not in the protocol	Yes, page 10
	20b	Methods for any additional analyses (eg, subgroup and adjusted analyses)	Yes, page 10
	20c	Definition of analysis population relating to protocol non-adherence (eg, as randomised analysis), and any statistical methods to handle missing data (eg, multiple imputation)	N/A
**Methods: Monitoring**			
Data monitoring	21a	Composition of data monitoring committee (DMC); summary of its role and reporting structure; statement of whether it is independent from the sponsor and competing interests; and reference to where further details about its	Yes, page 5

For peer review only

		charter can be found, if not in the protocol. Alternatively, an explanation of why a DMC is not needed	
	21b	Description of any interim analyses and stopping guidelines, including who will have access to these interim results and make the final decision to terminate the trial	N/A
Harms	22	Plans for collecting, assessing, reporting, and managing solicited and spontaneously reported adverse events and other unintended effects of trial interventions or trial conduct	Yes, page 5 (supervisory meetings) and page 11 (data protection)
Auditing	23	Frequency and procedures for auditing trial conduct, if any, and whether the process will be independent from investigators and the sponsor	Yes, page 5 (supervisory meetings) and page 11 (data protection)
**Ethics and dissemination**			
Research ethics approval	24	Plans for seeking research ethics committee/institutional review board (REC/IRB) approval	Yes, pages 2 and 11
Protocol amendments	25	Plans for communicating important protocol modifications (eg, changes to eligibility criteria, outcomes, analyses) to relevant parties (eg, investigators, REC/IRBs, trial participants, trial registries, journals, regulators)	N/A
Consent or assent	26a	Who will obtain informed consent or assent from potential trial participants or authorised surrogates, and how (see Item 32)	Yes, page 6
	26b	Additional consent provisions for collection and use of participant data and biological specimens in ancillary studies, if applicable	N/A
Confidentiality	27	How personal information about potential and enrolled participants will be collected, shared, and maintained in order to protect confidentiality before, during, and after the trial	Yes, page 11
Declaration of interests	28	Financial and other competing interests for principal investigators for the overall trial and each study site	Yes, page 12

For peer review only

Access to data	29	Statement of who will have access to the final trial dataset, and disclosure of contractual agreements that limit such access for investigators	Yes, page 11
Ancillary and post-trial care	30	Provisions, if any, for ancillary and post-trial care, and for compensation to those who suffer harm from trial participation	N/A
Dissemination policy	31a	Plans for investigators and sponsor to communicate trial results to participants, healthcare professionals, the public, and other relevant groups (eg, via publication, reporting in results databases, or other data sharing arrangements), including any publication restrictions	Yes, page 11
	31b	Authorship eligibility guidelines and any intended use of professional writers	Yes, page 11
	31c	Plans, if any, for granting public access to the full protocol, participant-level dataset, and statistical code	N/A
**Appendices**			
Informed consent materials	32	Model consent form and other related documentation given to participants and authorised surrogates	Yes, uploaded as supplementary material
Biological specimens	33	Plans for collection, laboratory evaluation, and storage of biological specimens for genetic or molecular analysis in the current trial and for future use in ancillary studies, if applicable	N/A

*It is strongly recommended that this checklist be read in conjunction with the SPIRIT 2013 Explanation & Elaboration for important clarification on the items. Amendments to the protocol should be tracked and dated. The SPIRIT checklist is copyrighted by the SPIRIT Group under the Creative Commons "Attribution-NonCommercial-NoDerivs 3.0 Unported" license.

For peer review only - http://bmjopen.bmj.com/site/about/guidelines.xhtml
